# Increased sensitivity of *BRCA* defective triple negative breast tumors to plumbagin through induction of DNA Double Strand Breaks (DSB)

**DOI:** 10.1038/srep26631

**Published:** 2016-05-25

**Authors:** Rakesh Sathish Nair, Jerald Mahesh Kumar, Jedy Jose, Veena Somasundaram, Sreelatha K. Hemalatha, Satheesh Kumar Sengodan, Revathy Nadhan, Thapasimuthu V. Anilkumar, Priya Srinivas

**Affiliations:** 1 Cancer Research Program, Rajiv Gandhi Centre for Biotechnology, Thiruvananthapuram, Kerala, India; 2 Centre for Cellular and Molecular Biology, Hyderabad, India; 3 Experimental Pathology, Sree Chitra Tirunal Institute for Medical Science and Technology, Thiruvananthapuram, Kerala, India

## Abstract

We have earlier shown that Plumbagin (PB) can induce selective cytotoxicity to BRCA1 defective ovarian cancer cells; however, the effect of this molecule in BRCA1 mutated breast cancers has not been analyzed yet. Here, we report that reactive oxygen species (ROS) induced by PB resulted in DNA DSB and activates downstream signaling by ATR/ATM kinases and subsequent apoptosis. PB reduces DNA- dependent protein kinase (DNA-PK) expression and inhibits NHEJ (Non Homologous End Joining) activity in BRCA1 defective breast cancer cells. Also, PB induces apoptosis in two different BRCA1 conditional knock out murine models: *MMTV-Cre; BRCA1*^*Co*/*Co*^ and *WAP-Cre; BRCA1*^*Co*/*Co*^, at 2 mg/kg body weight, but 32 mg/kg of carboplatin (CN) was needed to induce apoptosis in them. This is the first study where two different tissue specific promoter driven transgenic mice models with *BRCA1* exon 11 deletions are used for preclinical drug testing. The apoptosis induced by PB in HR (Homologous Recombination) defective triple negative BRCA1 mutant cell lines and in mouse models occur by inducing ROS mediated DNA DSB. The toxicity profile as compared with CN in transgenic mice provides evidence for PB’s safer disposition as a therapeutic lead in breast cancer drug development.

BRCA1 germline mutations have been identified in nearly 50% of hereditary breast cancers and 80% of cases with both hereditary breast and ovarian cancers. Furthermore, in sporadic breast cancers, *BRCA1* defects are seen due to its promoter hypermethylation or allele loss^1^. *BRCA1*/*2* deficient cancers mostly tend to be ER, PR and Her2 negative (triple negative) and are defective in HR repair machinery^2^. Initially these cancers tends to be sensitive to DNA cross linking agents, such as Cisplatin, CN and PARP inhibitors, resulting in an increased genomic instability, cell cycle arrest and apoptosis. However, restoration of BRCA1/2 function due to secondary mutations has been recognized as the mechanism for acquired resistance to Cisplatin and PARP (Poly (ADP-ribose) polymerase) inhibitors in these cancer cells^3^,^4^,^5^,^6^. BRCA1 mutated tumors are more sensitive to DSB inducing drugs.

Our group has earlier shown that antisense blocking of BRCA1 in BG1 ovarian cancer cells resulted in the induction of apoptosis in response to treatment with PB (2-hydroxy-5-methyl-1,4-naphthaquinone), a naphthaquinone isolated from plumbago plant species^7^,^8^,^9^. PB is known to generate ROS in cancer cells^10^. The ROS induced DNA damage may be irreparable in BRCA1 defective cells, as BRCA1 is involved in oxidative damage repair. However, a detailed mechanism of action of PB on DNA damage repair in BRCA1 defective triple negative breast cancer has not been analyzed till date.

ATM-dependent phosphorylation of H_2_AX (γH2AX) around the sites of DNA damage is one of the early events of DNA damage response . ATR and CHK2 are also phosphorylated in response to DNA damage. ROS generation can cause DNA damage which could induce the ATM mediated response^11^. The BRCA1 defective cancer cells show minimal level of HR or they are HR deficient. Hence, such ATM-dependent phosphorylation of H2AX around the sites of DNA damage is one of the early events of DNA damage response^12^ cells may rely primarily on NHEJ for DNA damage repair.

The present study intends to evaluate how the BRCA1 defect augments PB to selectively kill these cancer cells. Here, we provide evidence for the targeted anticancer activity of PB against BRCA1 mutated breast cancer cells *in vitro* as well as in GEMM (Genetically Engineered Mouse Models) of BRCA1 mutated mammary tumor. The ROS generated from PB induces DSB in these tumor and eventually lead to apoptotic cell death. The *in vivo* preclinical studies on anti-neoplastic function and toxicological evaluation of PB substantiate that it could be a potential candidate for monotherapy as well as combination therapy with PARP inhibitors or other standard chemotherapeutics for BRCA1-defective cancer treatment.

## Results

### Wild type BRCA1 reconstitution reduces the sensitivity towards PB in BRCA1 mutated breast cancer cells

**Figure 1:**
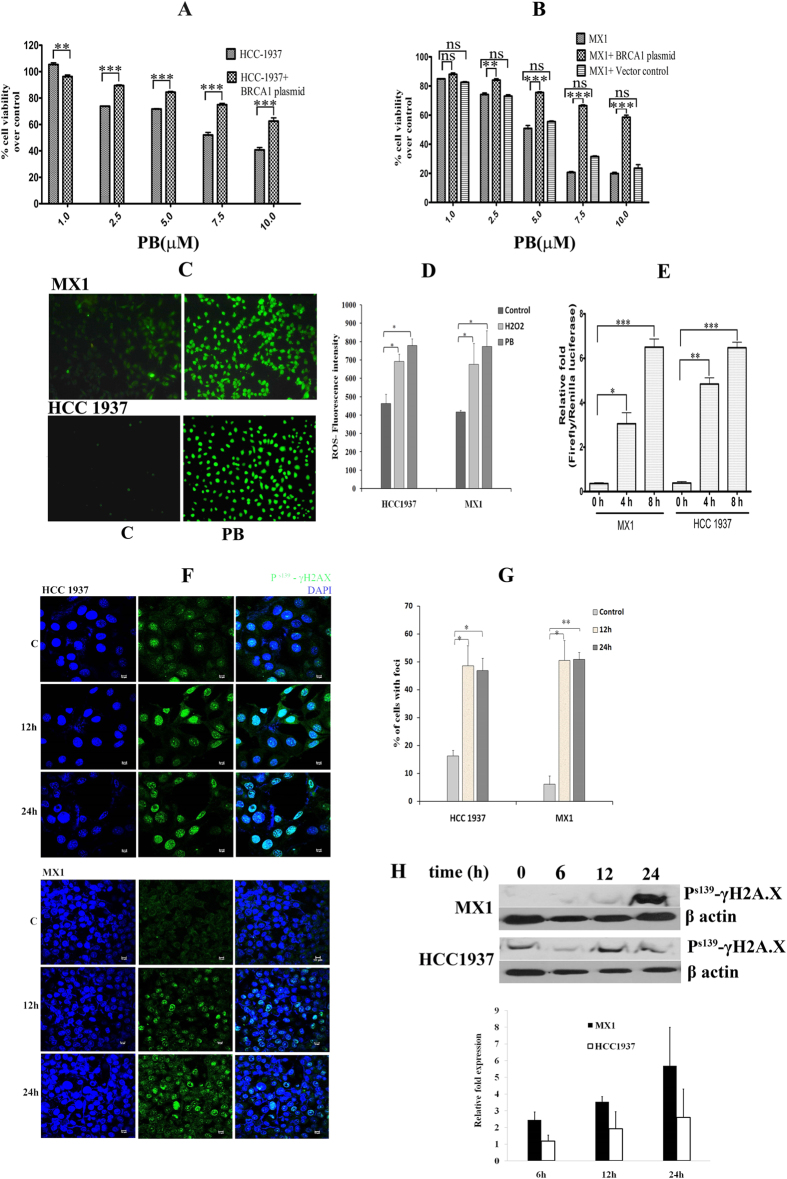
PB is less sensitive to BRCA1 reconstituted than BRCA1 mutated cancer cells. (**A**,**B**) MTT assay in MX1 and HCC 1937 cell lines reconstituted with wild type BRCA1 and vector control by PB treatment. (**C**) Reactive oxygen species induced after PB treatment and there by inducing antioxidant response elements. Cells were treated with PB for 4 h, washed with serum free medium, incubated with CM-H_2_DCFDA for 20 min and imaged in fluorescence microscope. The PB treated cells (PB) show bright green fluorescence than control (**C**) indicating the presence of ROS. (**D**) ROS measurement in PB treated cells by fluorimetric assay at 4 h. (**E**) The antioxidant response element Nrf1/2 expression using luciferase assay was performed after treatment with PB for 4 h and 8 h. p- values were calculated by comparing with the respective 0 h control (**F**). PB induces DSB in MX1 and HCC-1937. Immunocytochemistry revealed the expression of γH2AX in the form of foci , which directly correlates to the formation of double stranded breaks in cells treated with PB. The control cells showed low levels of green flourescence. Counter staining was performed using DAPI. (**G**) Number of foci formed by γH2AX expression quantified using Foci Counter software (**H**). Western blot was done with 50 μg protein from whole cell lysates. The chemiluminicent images and the quantification of bands represent the overexpression of γH2AX in both the cells which was significant at 24 h when compared to 0 h control (**C**). Lower panel shows the quantification of the above image which is a relative fold expression over 0 h control.

BRCA1 mutant, triple negative HCC1937 and MX1 breast cancer cells were used for the study. For anti-proliferative analysis, both HCC1937 and MX1 cells were reconstituted with wild type (full length) *BRCA1-GFP* plasmid and cytotoxicity of PB was analyzed by MTT assay. The IC_50_ value increased from 7.5 μM in HCC1937 cells to 12 μM in BRCA1 reconstituted HCC1937 cells (Fig. 1A). Similarly, in MX1 cells it changed from 4.5 μM to 9.8 μM (Fig. 1B). Hence, these data demonstrate that the ectopic expression of full length BRCA1 resulted in decreased sensitivity towards PB in BRCA1 mutated breast cancer cells. However, the transformed non-malignant breast cells MCF-10A, with wild type BRCA1, showed more resistance to PB with an IC_50_ of 16.57 μM; similar results were observed in a transformed normal breast cell line, HBL100 (Fig. S1). These results corroborate that PB induces cytotoxicity at a lower concentration in BRCA1 defective breast cancer cells in comparison to malignant as well as nonmalignant breast cells expressing wild type BRCA1.

### PB induced ROS causes activation of γH2AX

ATM-dependent phosphorylation of H2AX around the sites of DNA damage is one of the early events of DNA damage response^12^. PB treatment in MX1 and HCC1937 induced ROS, which was confirmed with CM-H_2_DCFDA (5-(and-6)-chloromethyl-2′,7′-dichlorodihydrofluorescein diacetate, acetyl ester) immunoflourescence staining (Fig. 1C). Quantitative fluorimetric analysis was also performed with H_2_O_2_ as positive control (Fig. 1D). The antioxidant response element *Nrf1*/*2* showed a 6 fold over expression in MX1 and HCC1937 with PB (Fig. 1E). When treated with PB, the number of γH2AX foci was more (Fig. 1F,G) in MX1 cells and was significantly higher when compared to HCC1937, which was confirmed by western blot (Fig. 1H). Thus, PB induces ROS, resulting in the expression of antioxidant response element *Nrf1*/*2;* however, defective HR due to absence of wild type BRCA1 might still cause DNA DSB.

### PB inhibits the NHEJ activity and down regulates the DNA-PK expression

**Figure 2:**
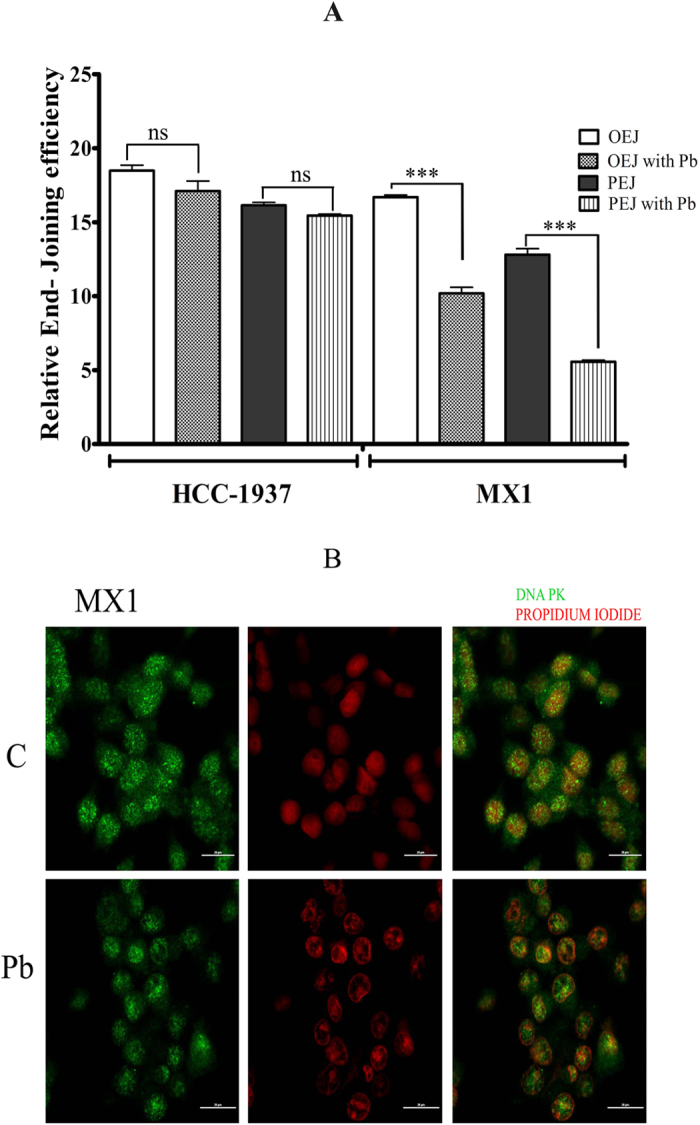
PB inhibited NHEJ and DNA-PK in MX1 but not in HCC 1937. (**A**) The relative end joining efficiency was calculated from the luciferase activity after treating with PB for 6 h. The differences among the mean values were analyzed using One-way ANOVA followed by bonferroni post hoc t- test (OEJ -Overall end joining; OEJ with Pb- Overall end joining in the presence of PB; PEJ- Precise end joining; PEJ with Pb- PEJ in the presence of PB) (**B**). The Immunoflourescence analysis with DNA-PK antibody on MX1 cells treated with PB compared to untreated control (**C**). The counter staining was performed using nuclear stain Propidium Iodide.

To assess the NHEJ, a luciferase-based plasmid repair assay was performed. In brief, a cut was introduced in the luciferase plasmid (pGL2), which was then transfected into the cells and repair via NHEJ was measured by relative luciferase activity. The end-joining capacity detected using pGL2 digested with HindIII, reflects overall end-joining because this enzyme cleaves at the linker region between the promoter and the coding sequences, and any end-joining activity, even that resulting from small deletions or insertions, would not affect the luciferase expression. However, as the EcoRI site is in the luciferase sequence and only precise end-joining (PEJ) would restore the original luciferase action, the relative luciferase activity using this enzyme reflects PEJ capacity^13^. We have employed this plasmid based luciferase repair assay to see whether PB (ROS inducer) can inhibit NHEJ. PB treatment resulted in a significant reduction in PEJ activity and OEJ (Overall end-joining) activity (P < 0.001) with PB treatment in MX1 cells. Though PEJ and OEJ are reduced in HCC 1937, the reduction was not significant (Fig. 2A). Since, the DNA damages induced by PB is not repaired either by HR or NHEJ, the cells will be more sensitive to PB leading to apoptosis. DNA-PK, plays a pivotal role in NHEJ pathway. The activated DNA-PK has serine/threonine kinase activity that is necessary for efficient repair^14^. MX1 cells treated with PB showed reduced expression of DNA-PK with a fuzzy foci formation when compared to the control cells (Fig. 2B). Thus, the decreased NHEJ activity may be due to the reduction of DNA-PK foci formation.

### PB induces DNA damage response via phosphorylated ATR, ATM and Chk1/2

**Figure 3:**
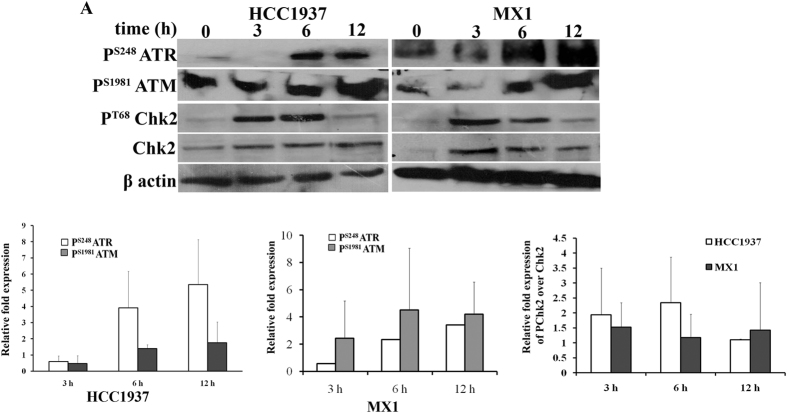
Over expression of phophorylated ATR and Chk2 on PB treatment in MX1 and HCC1937 cells. (**A**) Cells were treated with PB for 3, 6 and 12 h. Expression for phospho ATM, ATR and Chk2 and non-phosphorylated Chk2 were performed using western blotting. The expression of phosphorylated Chk2 was elevated while non phosphorylated form does not show any difference in the expression. (**B**) Quantification of the western blot has been shown in the lower panel.

Phosphorylated forms of ATR (P^S428^-ATR) and ATM (P^S1981^ ATM) are expressed in cancer cells as a result of DNA damage. In both HCC1937 and MX1 cells, PB increased the expression of phosphorylated ATR, ATM and Chk (Fig. 3A,B), indicating the activation of DNA damage response pathway.

### PB activates p53, p21 and PARP in *BRCA1* mutated breast cancer cells

**Figure 4:**
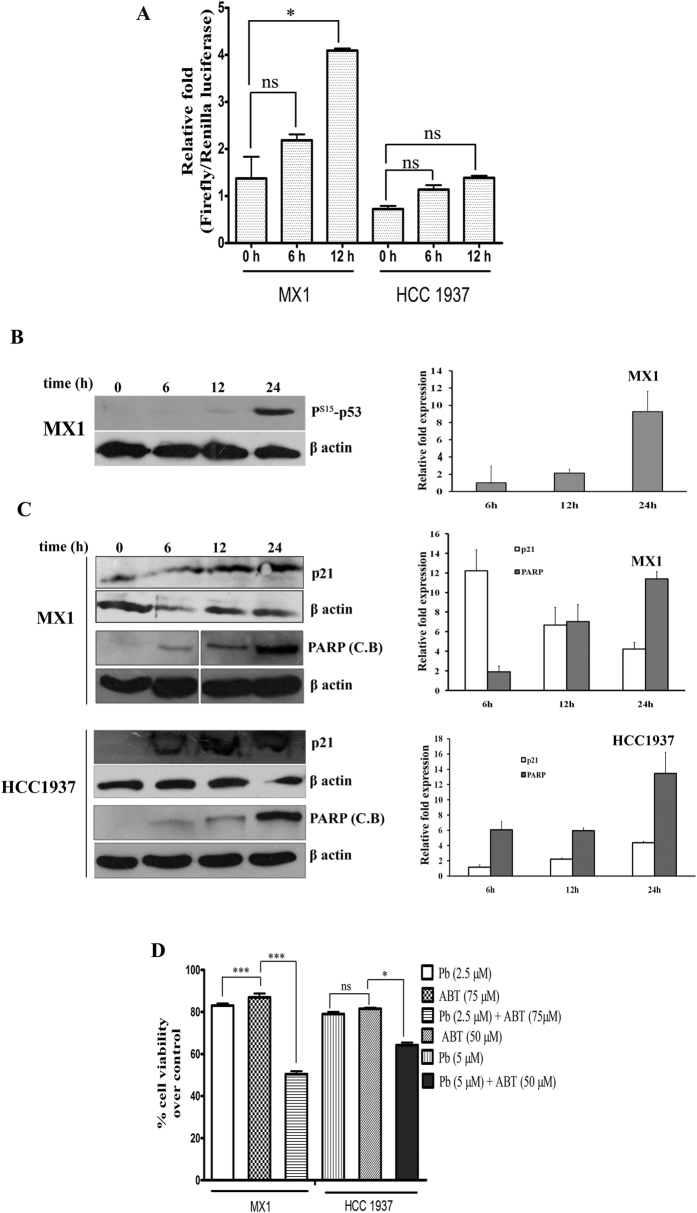
(**A**) Differential expression of p53 in BRCA1 mutated breast cancer cells. The luciferase reporter assay was performed after treatment with PB for different durations as represented in graph. P- values were calculated by comparing with the respective 0 h control. (**B**) Western blot data showing that phophorylated p53 was over expressed due to PB treatment in MX1cells. Quantification of western blot has been shown in the right side of western blot image. (**C**) Proteins related to apoptosis are expressed on PB treatment. PB show over expression for apoptotic proteins, in MX1 and HCC 1937 cells: p21 and PARP cleaved band (**C**.B.) overexpression was observed. Quantification of western blot has been shown in the right side of western blot image. (**D**) Analysis of cell viability in PB and ABT-888 treated cells by MTT assay. BRCA1 mutated breast cancer cells were more sensitive to the combination of PB and ABT-888 rather than single but MX1 showed effective reduction in viability when compared to HCC-1937. Cells were treated with or without combination of indicated concentration of drugs for 48 h. At the end of treatment, cell viability was assessed by MTT assay as described in materials and methods. All results are expressed as the mean percentage of control +/− S.D. of quadruplicate determinations from three independent experiments. The differences among the mean values were analyzed using One-way ANOVA followed by bonferroni post hoc t- test by comparing with respective single drug (Pb- PB and ABT- ABT-888; the parenthesis represents the concentration in μM).

Using p53-responsive luciferase reporter assay, it has been observed that there was a 3 fold over expression of p53 transcripts in MX1 cells. However, the increase was not significant in HCC1937 probably because p53 is mutant in these cells (Fig. 4A). PB induced the expression of phosphorylated form of p53 (P^S15^-p53) in MX1 cells (Fig. 4B). The p53 transactivated protein, p21, showed an increased expression after PB treatment in both MX1 and HCC1937 cells (Fig. 4C). PB treated MX1 showed expression of the cleaved PARP band (89 kDa) at 12 h, while in HCC1937, the maximum expression was observed at 24 h (Fig. 4C).

### PB in combination with PARP inhibitor (ABT-888) elicits improved cytotoxicity

PARP inhibitors are proved to be effective against BRCA1/2 mutated cancers and several phase III clinical trials are currently going on. The cytotoxicity of PARP inhibitor, ABT-888 in combination with PB was analyzed in MX1 and HCC1937 cell lines. The IC_20_ concentration (~80% cells are viable) of PB (2.5 μM) and ABT-888 (75 μM) were used individually or in combination in MX1 cells (Fig. 4D). The combination reduced the viability to 50% with a Combinatorial Index value (CI < 0.85) which showed moderate synergism in MX1 cells. Similarly, in HCC1937 cells, the combination of IC_20_ concentration of PB (5 μM) and ABT -888 (50 μM) reduced the viability to 65% with a CI value > 1.45. Thus, in combination with PB, ABT-888 showed increased cytotoxicity against BRCA1 mutated breast cancer cells.

### PB induces apoptosis in mammary glands of *MMTV-Cre; BRCA1* ^*Co*/*Co*^ & *WAP-Cre; BRCA1* ^*Co*/*Co*^ mouse

**Figure 5:**
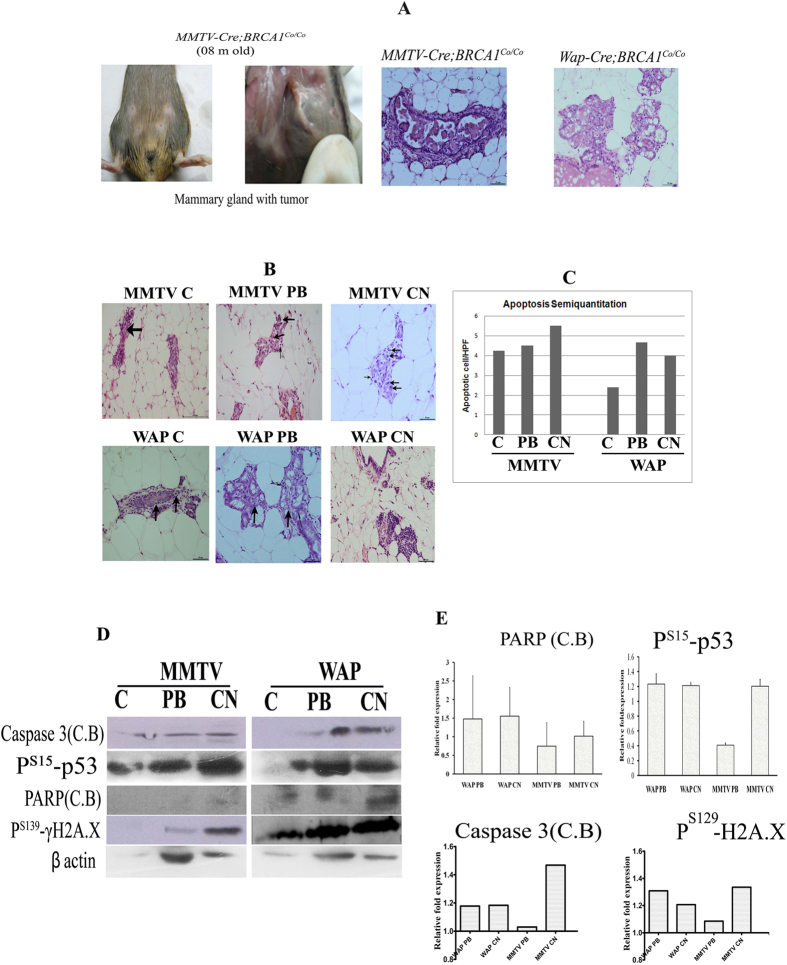
(**A**) WAP-Cre; BRCA1^Co/Co^ and MMTV-Cre; BRCA1^Co/Co^ conditional knockout form mammary specific tumors of longer latency. Gross appearance of mammary gland representing tumor in MMTV-Cre; BRCA1^Co/Co^animals. H&E staining representing tumorigenic lesions in MMTV-Cre; BRCA1^Co/Co^ & WAP-Cre; BRCA1^Co/Co^ animals. (**B**) Cell death by necrosis/apoptosis in the mammary glands of WAP-Cre; BRCA1^Co/Co^ and MMTV-Cre; BRCA1^Co/Co^ animals in the PB and CN treated group. Panel (Top row) MMTV C show moderate hyperplasia of the ductal cells with mild cellular atypia and occasional mitosis (arrow); MMTV PB with moderate cellular atypia of ductal cells with more frequent mitosis (thick arrow) and apoptosis (thin arrow) than in MMTV C group while in MMTV CN the mammary gland had moderate cellular atypia of ductal cells with more frequent mitosis (thick arrow) and apoptosis (thin arrow) than in MMTV PB group. Moderate cellular atypia of ductal cells with more frequent mitosis (thick arrow) is seen in WAP C group (bottom row) while the WAP PB had moderate cellular atypia of ductal cells with more frequent mitosis (thick arrow) and apoptosis (thin arrow) than in WAP C group but the histology section of WAP CN show multifocal lymphoid aggregates in mammary gland. (**C**) The graph represents the percentage of apoptotic cells of the H & E section. (**D**) Apoptotic proteins are expressed in the mammary tumors of WAP-Cre; BRCA1^Co/Co^ and MMTV-Cre; BRCA1^Co/Co^ when treated with PB. Western blotting was performed in lysates from mammary tumors. Caspase 3 cleaved (C.B.) protein, phosphorylated p53, PARP and γH2A.X) showing differential expression in mouse mammary tissue due to PB treatment. (**E**) The right panel shows the quantification of the western blot. The WAP and MMTV group that were treated with PB are annotated as WAP PB and MMTV PB; CN treated group annotated as WAP CN and MMTV CN; solvent treated control group as WAP C and MMTV C respectively.

The mouse model used in the study is the female F1 progeny of BRCA1 floxed female mice and MMTV-Cre or WAP-Cre male mice produced by utilizing the CRE-LOX recombination system. Since, BRCA1^Co/Co^ animals lack BRCA1 exon 11 it will generate triple negative tumors as reported earlier^15^. In our animal models, conditional disruption of BRCA1 results in genomic instability, which would cause genetic alterations including the inactivation of p53 in the breast tissue, leading to tumor formation^16^. The *MMTV-Cre; BRCA1*^*Co*/*Co*^ and *WAP-Cre; BRCA1*^*Co*/*Co*^ animals were physically examined for palpable tumors. Tumors in *WAP-Cre; BRCA1*^*Co*/*Co*^ animals were confirmed using *in vivo* NMR bio-imaging (Fig. S2). NMR density in the mammary area indicated the tumor growth. C57B/L of same age group was used as control. Necropsy conducted in *MMTV-Cre; BRCA1*^*Co*/*Co*^ and *WAP-Cre; BRCA1*^*Co*/*Co*^ mouse, followed by histopathology (Fig. 5A) confirmed the neoplastic nature of the mammary gland tissue. All animals were treated with PB, CN and the solvent treated control group had hyperplastic ductal epithelium with moderate dysplasia in the breast tissue. The lesions detected in histopathology were classified as ductal carcinoma as per Cardiff’s criteria^17^. Mitotic figures were frequently seen in the mammary duct-lesions along with cell death by apoptosis in PB and CN treated animals as compared with control animals in both experimental strains (Fig. 5B). Quantification of apoptotic cells has been shown in Fig. 5C. In addition, the WAP-CN group had multifocal lymphocytic infiltrations, which were disorganized lymphoid follicles with frequent mitosis.

### PB induces phosphorylated-p53 and γH2AX in tumors of *WAP-Cre; BRCA1*^*Co*/*Co*^ animals

PB treatment in mammary tumor of both *WAP-Cre; BRCA1*^*Co*/*Co*^ and *MMTV-Cre; BRCA1*^*Co*/*Co*^ animals showed increased expression of both phosphorylated p53 (P^S15^-p53) and γH2AX. WAP animals showed higher expression of γH2AX when treated with PB than CN. However, in MMTV group, the γH2AX and P^S15^-p53 expression were less, when treated with PB than with CN (Fig. 5D,E).

### PB induces the Caspase 3 and PARP proteins in mammary tumor

Histopathologically, there were abundant apoptotic lesions due to drug treatment and hence, the expression of selected apoptotic markers was validated in the mammary tumors. Activated Caspase 3 and PARP expression were similar in both PB and CN treated WAP animals (Fig. 5D,E). In MMTV animals, CN showed increased expression than PB for activated caspase 3 and PARP cleaved band. Thus, *in vivo* studies showed that WAP animals were more sensitive to PB than CN, while MMTV animals were more sensitive to CN.

### Toxicological analysis of PB in *WAP-Cre; BRCA1*^*Co*/*Co*^ and *MMTV-Cre; BRCA1*^*Co*/*Co*^ animals

The body weight was monitored once in every seven days in all animal groups and treatment with PB/CN did not cause significant change in the body weight (Fig. S3A). The overall toxicological evaluation was performed in all the organs in each group. The toxicological evaluation revealed that the CN treated group of both WAP and MMTV were susceptible to cardiac toxicity. CN is second generation platinum containing anticancer drug and it has been found that it can induce cardiotoxicity via mitochondrial pathway. Lesions in the heart, such as occasional degeneration of myocardiocytes and mild hemorrhage, that were observed might be due to congestion of the cardiac tissue in all groups irrespective of the treatment and were considered as background lesions by the pathologist. However, focal necrosis and focal myocarditis were present in WAP-PB group and WAP-CN group respectively. MMTV-CN had mild hypertrophy. There was no unique lesion in the heart of the PB treated animals. PB did not show any significant nephrotoxicity while CN in both groups showed proliferative glomerular nephritis and multifocal nephrosis with moderate hypertrophy of the afferent arteriole. Glomerular lesion in MMTV CN was significant compared to other groups with moderate thickening of the Bowman’s capsule. Moderate hypertrophy of hepatocytes and mild to moderate congestion of liver were present in all the specimens. A solitary clear cell focus and microgranuloma was present in MMTV CN and WAP CN respectively (Fig. S3B). Spleen was generally free of lesions in all MMTV-groups but there was mild hyperplasia of lymphoid follicles in WAP-animals treated with both PB and CN. The histomorphology of uterus reflected only the changes in physiological status. Mild hyperplasia was seen in most samples. Occasional sub endometrial infiltration of mononuclear cells (MMTV-C) or neutrophils in WAP-PB were present (data not shown). The brain was free of toxicological lesions. Thus, it can be concluded that PB is non-toxic at this particular dosage, but need more investigations to define the optimum dose for clinical use.

## Discussion

Currently, an effective treatment regimen targeting BRCA1-defective breast cancers is absent. Sensitivity of BRCA1 deficient cells to doxorubicin is highly controversial^9^,^18^,^19^, while cisplatin showed reversal mutations following treatment in BRCA1 mutated tumors owing to its increased resistance^4^,^20^.

Anti-cancer effects of PB have been reported in diverse cancer models such as prostate^21^, lung^22^,^23^, cervical^10^,^24^ ovarian^8^ melanoma^25^ and breast cancer^26^. The effect of PB against BRCA1 mutated ovarian cancers were previously reported from our laboratory with evidences directing to the modulation of Estrogen Receptor (ER) α, a 46 kDa isoform, playing a critical role in blocking the classical ER signaling in ER over expressing ovarian cancer cells^7^,^8^,^9^. However, in the case of triple negative breast tumors, the signaling pathway of PB induced apoptosis may be different. In this study, the cytotoxic potential of PB was evaluated in triple negative (ER, PR and Her2 negative), BRCA1 defective breast cancers since majority of the BRCA1 associated cancers belong to this category. Here, reconstitution of wild type BRCA1 plasmid in both MX1 and HCC1937 showed selective cytotoxic activity of PB against BRCA1 mutated cancer cells. Non-toxic activities of PB in normal transformed cells were earlier reported in MCF-10 A and HBL-100 breast cells^24^,^27^ and in non-tumorigenic immortalized prostate epithelial RWPE-1 cells^28^.

ROS generation by PB was reported earlier in various cancers^7^,^10^,^29^. Nrf2 and Nrf1 bind to the antioxidant response elements (AREs) and regulate genes involved in protecting the cells from oxidative damage. MX1 and HCC1937 cells show ROS generation and enhanced transcriptional activity of Nrf1/2 of ARE in the presence of PB. It has also been reported earlier that PB increases nuclear localization and transcriptional activity of Nrf2, and induces the expression of the Nrf2/ARE dependent genes^30^. The elevated expression of haemoxygenase, which is an Nrf1/2 dependent gene, as a result of PB treatment in BRCA1 blocked BG1 ovarian cancer cell has also been reported earlier by our own group, confirming the activation of the antioxidant mechanism^9^.

PB treatment induces the phosphorylation of H2AX in HCC1937 and MX1 cells which is a hallmark of DNA DSB. Thus, a simultaneous activation of antioxidant mechanism along with DNA DSB was observed by PB treatment. The cellular antioxidant mechanism is getting activated as a consequence of protecting the cells against ROS insult. However, DNA DSB occurs in BRCA1 defective cancer cells which might be either due to the insufficient antioxidant mechanism or a higher quantum of ROS.

As in MCF-10 A, where BRCA1 is wild type, intact γH_2_AX foci formation could not be observed in BRCA1 defective cells. A fuzzy appearance of γH_2_AX foci formation has already been reported in HCC1937 cells compared to MCF10A in response to irradiation^31^. γH_2_AX foci formation in response to DNA DSB occurs before BRCA1 induction, so it should not be hindered even if BRCA1 is defective. We have observed over expression of γH_2_AX protein by PB treatment in both the cell lines and the mouse tumor tissues. Therefore, there could be defect in the nuclear import of γH_2_AX, even when the protein is sufficiently produced in the cells in response to DNA damage, resulting in impaired formation of foci in the nucleus in a BRCA1 defective condition.

BRCA1 primarily acts at the proximal step that signals the presence of DSB and helps to initiate their repair by HR, whereas BRCA2 stabilizes the structure of replication-associated lesions and works directly to resolve them using HR, by controlling the activity and assembly of the essential recombination enzyme RAD51^32^. PB treatment causes inhibition of NHEJ in both the cell lines, but was more evident in MX1 cells while in HCC1937, the extent of repair inhibition was low which might be due to reduced DNA-PK inhibition after PB treatment. The exact reason for this difference in the NHEJ activity between the two cell lines is not known. Since MX1 cells are deficient in both BRCA1 and BRCA2, it may have defective mitotic spindle assembly check point. Mechanistically, when NHEJ was inhibited there was a compensatory increase in HR^33^. Since, these cell lines were HR deficient, DNA damage persists resulting in accumulation of mutations and induction of apoptosis. In such cases, cells may become sensitive to DNA damaging agents. This also points out the ability of PB to sensitize BRCA1-defective cancer cells to DNA damaging drugs or radiation. The radio sensitization ability of PB has already been reported in cervical cancer cells^24^.

ATM and ATR, play a central role in DNA damage recognition and the initial phosphorylation events required for the commencement of the repair process in association with their respective effectors, Chk2 and Chk1^34^. It was reported that PB treatment resulted in the enhanced activation of Chk2 by suppressing Akt pathway^26^. We have shown that PB increased phosphorylation of ATM and ATR, thereby inducing phospho-Chk2 levels in BRCA1 mutated cells leading to further downstream cellular events culminating in apoptosis.

DNA damage response causes p53 phosphorylation which further leads to cell cycle arrest by inducing p21 and PARP. In this study, we have observed that PB can induce phospho p53 (p^S15^-p53) expression in MX1 cells but not in HCC1937 cells, possibly due to the presence of wild type p53 in MX1, while it was mutated in HCC1937^35^,^36^. PB treatment results in p21 induction in both the cell lines indicating that p21 induction is p53 dependent in MX1 cells and p53 independent in HCC1937 cells.

MX1 cells have wild type p53, where as it is mutant in HCC1937. p53 protein interacts at the C-terminal region of BRCA1 and promotes the export of BRCA1 from nucleus to cytoplasm^37^ so that it is not available for DNA damage repair. Such cells will be more sensitive as it induces p53 mediated apoptosis. p53 defective cells cause retention of BRCA1 in the nucleus, resulting in the repair of the DNA damages and thus will be resistant to drugs. However, since both MX1 and HCC1937 lack wild type BRCA1, there could be inhibition of DNA damage repair. But since MXI has wild type p53, BRCAI independent p53 mediated apoptosis could happen in these cells. It has been reported that FOXO3 interacts with the ATM-Chk2-p53 complex directly, and trigger apoptosis as a result of DNA damage^38^. BRCA1 is not needed for this apoptosis mechanism. Thus, in both MX1 and HCC1937 apoptosis could be induced by DNA damaging agents, particularly by oxidative damage inducers like PB, as BRCA1 is needed for oxidative damage repair also. BRCA1 regulates transcription coupled repair of 8 - Oxoguanine (8 -oxoG) DNA lesion caused by oxidative stress through base excision repair. Therefore, apoptosis induction through cellular ROS production may be an effective strategy to treat BRCA1 associated cancers^39^.

PARP is involved in the repair of single strand DNA breaks, and inhibition of the enzyme results in an impairment of DNA repair resulting in increase in the number of double-strand DNA breaks. This phenotype is particularly detrimental to cells with no intact BRCA1 or BRCA2 protein and results in cell death. The use of PARP-1 inhibitors in combination with standard chemotherapeutic agents also, seems attractive in the sense that sensitizing tumor cells to cytotoxic agents might enable the use of lower doses, while maintaining the same relative efficacy with reduced toxic side effects. PB induces PARP activation; therefore, the cytotoxicity of PB will be more when used in combination with PARP inhibitors. The combination of PB with PARP inhibitor (ABT-888) in both MX1 and HCC1937 cells proved their synergistic activity by reducing the cell viability to 62.5% and 75% respectively. The combination of PARP inhibitor, ABT-888, has been shown to potentiate anticancer activity of topotecan and cisplatin^40^,^41^.

In order to assess the *in vivo* activity of PB, it was tested in a transgenic model. The *WAP-Cre; BRCA1*^*Co*/*Co*^ and *MMTV-Cre; BRCA1*^*Co*/*Co*^ animal models developed triple negative breast cancers with a basal-like phenotype that exhibited a gene expression pattern paralleling human breast cancers^16^,^42^. Conditional transgenic models of BRCA1 mutant breast cancers have not been used widely in preclinical trials except for the study of *K14-Cre; BRCA1*^*fl*/*fl*^ mouse in testing the efficacy of Olaparib^43^ and Topotecan^44^, wherein BRCA1 5-14 exon floxed animals were used.

Histological analysis of apoptotic cells and expression pattern of caspase 3, PARP, γH_2_AX and phosphorylated p53 showed that *WAP-Cre; BRCA1*^*Co*/*Co*^ animals were more sensitive to PB while *MMTV-Cre; BRCA1*^*Co*/*Co*^ animals were more sensitive to CN. Since, MMTV is a promoter which is expressed in many organs of the animal; there is no specific recombination event which is considered to be organ (breast) specific which is analogous to the episode taking place in WAP -Cre F1 chimera. However, we do not have an explanation on why WAP based tumors respond more to PB than MMTV based tumors. The dosage of 2 mg/kg b.w. was sufficient to elicit apoptosis induction by PB, while 32 mg/kg b.w. of CN was required for comparable activity.

PB has been reported to be non-toxic at concentrations (2 mg/kg b.w.) that we have used in this study^45^,^46^,^47^. In our study, signs of toxicity were absent, as judged by parallel monitoring of body weight and histological analysis of heart, kidney, ovary and brain in PB treated mice.

In conclusion, PB induces DSB in DNA, triggers downstream signaling by expression of γH2AX and ATR/ATM kinases, and inhibits NHEJ resulting in p21 induced apoptosis. Cells having functional BRCA1, including normal cells, resist the DNA damage due to the presence of a functional HR/NHEJ repair mechanism. The study involving the combination of PB with PARP inhibitors facilitate the therapeutic repositioning of PARP inhibitors in effective inhibition of cancer cell growth. In our study, the concentration of PB administered was sixteen times less than that of CN. We have seen that CN could cause mild toxicity to heart, liver and kidney. CN is also reported to induce side effects including peripheral neuropathy, central neurotoxicity, nephrotoxicity, ototoxicity and even abnormal cardiovascular events. The present study reveals that PB will not elicit any toxicity of to the normal tissues *in vivo*. Thus, the current study provides the foundation for the future use of PB for properly scheduled phase trials for the treatment of BRCA defective cancers.

## Methods

### Cell lines used

Human breast ductal carcinoma cell lines HCC1937 (5382insC) and MX1 having mutations in both BRCA1 (3363delGAAA) and BRCA2 (16864A > C and BRCA2 221847A > G) were used for the *in vitro* study. The details of the cell lines are given in the supplementary materials.

### *In vitro* cell viability assay

The cell viability studies were performed using a colorimetric MTT assay which is described in supplementary material.

### ROS induction

The ability of PB to induce ROS production was assessed in MX1 and HCC1937 cells by CM-H_2_DCFDA which gets oxidized to bright green colored DCF by ROS and the fluorescence was measured microscopically. The quantitative flourimetric analysis was also performed. Briefly, cells grown in 96 well plates were washed with PBS and incubated with CM-H_2_DCFDA for 30 minutes at 37 °C in dark. Then these cells were treated with PB and 10 μM H_2_O_2_ as positive control, for 4 h. ROS generation was measured using fluorescence microplate reader with an excitation wavelength of 488 nm and emission wavelength of 535 nm (TECAN infinite 200).

### Reporter assay for Nrf 1/2 and p53 promoter activity

Assay procedure is described in the supplementary section.

### Immunoflourescence

MX1 and HCC1937 cells were treated with PB for 12 h and 24 h and flourescence imaging was performed for phosphorylated (P^S139^) H2AX (γ-H2AX) protein. For DNA-PK immunofluorescence cells were treated for 12 h. Cells counterstained either with 0.5 μg/ml DAPI or Propidium Iodide (PI) for 15 min, mounted in Prolong anti-fade reagent (Life Technologies, NY, USA) and imaged using confocal microscope. Image acquisition and foci counting were performed using Foci Counter program.

### Western blotting

Cell lysates were isolated from cultured cells as well as mammary tumor tissues and western blot analysis for various proteins was performed as detailed in the supplementary section.

### *In vivo* end-joining assay

The details of *In vivo* end joining assay are provided in the supplementary material.

### Animal experiments

Animals strains used in this study are WAP-Cre mice [STOCK 01XA8, B6.Cg-Tg (Wap-Cre) 11738Mam], MMTV-Cre mice [STOCK 01XA9, B6.Cg-Tg (MMTV-Cre) FMam] and BRCA1floxed mice [STOCK 01XC8 Brca1tm1Cxd], obtained from the NCI mouse repository at National Cancer Institute (NCI), USA. The generation of *WAP-Cre; BRCA1*^*Co*/*Co*^ and *MMTV-Cre; BRCA1*^*Co*/*Co*^ conditional knockout mouse models and the experiments with PB and CN are described in the supplementary material. The *in vivo* studies were performed in accordance with the approved guidelines of Institutional Animal Ethical Committee, Centre for Cellular and Molecular Biology (CCMB), Hyderabad, India.

### Histopathology by H & E staining

Details of the histological study are provided in the supplementary material.

### Statistical analysis

The independent-sample t-test was used to test the probability of significant differences between different experimental groups. ANOVA followed by Bonferroni’s post hoc test was used for multiple comparisons between multiple groups. Statistical significance was defined as *P ≤ *0.05;* **P ≤ *0.001;* ***P ≤ *0.0001* and “ns” denotes for non-significance. Error bars were given on the basis of calculated S.D values. All statistical analysis was performed using GraphPad Prism™ trial version for Windows (GraphPad Software, San Diego, California, USA).

## Additional Information

**How to cite this article**: Nair, R. S. *et al.* Increased sensitivity of *BRCA* defective triple negative breast tumors to plumbagin through induction of DNA Double Strand Breaks (DSB). *Sci. Rep.* **6**, 26631; doi: 10.1038/srep26631 (2016).

## Supplementary Material

Supplementary Information
