# Supplementary material for: Increased sensitivity of BRCA defective triple negative breast tumors to plumbagin through induction of DNA Double Strand Breaks (DSB)
Source: Sci Rep. 2016 May 25;6:26631. doi: 10.1038/srep26631 (PMC4879579; doi:10.1038/srep26631)
Supplement: Supplementary Information [file srep26631-s1.doc]

**Increased sensitivity of *BRCA* defective triple negative breast tumors to plumbagin through induction of DNA Double Strand Breaks (DSB)**

Rakesh. S. Nair^1¶^, Jerald. M. Kumar^2^, Jedy Jose^2^, Veena Somasundaram^1¶¶^, Sreelatha K. Hemalatha^1^, Satheesh Kumar S^1^, Revathy Nadhan^1^, Anilkumar Thapasimuthu^3^ and Priya Srinivas^1*^

**Supplementary Materials and Methods**

**Reagents**

**Cell lines used**.

HCC1937 (5382insC mutation that leads to the synthesis of a C-terminal truncated BRCA1 protein) were kind gifts from Dr. Grant Mc Arthur, Peter MacCallum Cancer Centre, VIC, Australia. MX1 breast carcinoma cell line was derived from poorly differentiated mammary carcinoma. These cells were provided as a free gift from Dr. Cathrin Dressler, Laser- und Medizin-Technologie GmbH, Berlin, Germany. Novel BRCA1 variant (BRCA1 3363delGAAA) was detected that would result in a frame shift mutation predicted to introduce a chain terminator and truncate the protein at residue 999 [29]. Two non synonymous single nucleotide polymorphisms were detected in BRCA2 (BRCA2 16864A>C and BRCA2 221847A>G) also in MX1 cell line. Cell lines were triple negative (ER, PR and Her2/neu negative). The cells were grown in RPMI-1640 with 10% FBS.

***In vitro* cell viability assay.**

Invitro cell viability, MTT assay was performed in 96 well plate (6000 cells/well) and incubated for 48 h with or without PB. After incubation, cells were treated with, 100 μl of MTT working solution (1 mg/ ml prepared in 2.5% culture medium) and incubated for 1.5 h at 37^o^C. Then, 100 μl of lysis buffer (20% Sodium Dodecyl Sulphate in 50% Dimethyl Formamide) was added to the wells and incubated at 37^o^C further for 2.5 h. Absorbance was measured using a microplate reader (Bio-Rad Laboratories, Hercules, CA, USA). The relative cell viability is expressed as (Abs_570_ of treated wells/ Abs_570_ of untreated wells) X 100.

**Reporter assay for Nrf 1/2 and p53 promoter activity.**

The reporter assay was performed using Cignal p53 Reporter Assay kit (SABiosciences, QIAGEN Inc., Valencia, CA). The ARE reporter is a mixture of a Nrf1/2 responsive luciferase construct and a constitutively expressing Renilla construct. The p53-responsive luciferase construct encodes the firefly luciferase reporter gene under the control of a minimal (m) CMV promoter and tandem repeats of the p53 transcriptional response element (TRE).  The transfection is performed in 24 well format using the forward transfection procedure with the help of SureFECT transfection reagent (SABiosciences, QIAGEN Inc., Valencia, CA, USA) as per manufactures protocol. Briefly, 250 ng of plasmid construct is used per well and and incubated for 16 h post- transfection in fresh medium and with drugs for next 32 h. Finally the luciferase assay is performed using Dual Luciferase assay (Promega Corporation, Madison, WI, USA) in a Luminometer.

***In Vivo* End-Joining Assay**

The role of PB in DSB repair by NHEJ is examined in HCC1937 and MX1 using a plasmid end-joining assay. *In vivo* end joining assay are performed as per the protocol sited by Bau et al., 2004. The assay is performed using pGL2 plasmids. In short plasmid pGL2 is completely linearized using either *Hin*dIII or *Eco*RI, as confirmed by agarose gel electrophoresis. The linearized DNA is subjected to phenol/chloroform extraction. Ethanol-precipitated DNA is dissolved in sterile water, and transfected to cells using Lipofectamine 2000 (Life Technologies, NY, USA) as per the manufacturer's protocol. 36 h post transfection cells are treated with PB for 6 h. The transfectants are harvested and assayed for luciferase activity. The cell lines were treated with PB with a concentration less than IC_50_, [MX1 (2.5 μM) and HCC1937 (5 μM)]. Repair efficiency is calculated from the luciferase activity of linearized reporter constructs compared with that of the intact plasmid. The NHEJ activity of a cell is the sum of Overall End Joining (OEJ) and Precise End Joining (PEJ).

**Extraction of total Protein from Tissue Samples and Western blotting**

Mammary tumor tissue was isolated and collected in All Protect Tissue Reagent were thawed on ice for 30 min and weighed. Approximately 250 mg tissue was used to isolate the total protein. Western blot analysis was performed to detect the expression levels of γH2AX, p21, PARP in MX1 and HCC1937 cell lysates after 0, 6, 12 and 24 h treatments with PB. P^S15^-p53, Caspase 3, PARP and γH2AX were also analysed in tumour tissue lysates from BRCA1 knockout mice. β-actin was employed as the loading control. The tissue lysates were prepared with the mammary gland lysis buffer (50 mM Tris, pH 8.0, 300 mM NaCl, 20% Glycerol and Protease Inhibitor cocktail), while the MX1 and HCC1937 whole cell lysates were prepared in RIPA (Radio Immuno Precipitation Assay) buffer supplemented with protease inhibitor cocktail and with or without phosphatase inhibitor. Supernatant was collected by centrifugation at 13,000 rpm for 10 min. The whole cell lysates and tissue lysates were then subjected to sodium dodecyl sulphate polyacrylimide gel electrophoresis (SDS-PAGE) and then transferred to Nitrocellulose membranes and immunoblotted. The proteins bound to specific antibodies were detected using Enhanced Chemiluminescence (ECL, Amersham, Piscataway, NJ).

**BRCA1 conditional knockout mouse models**

**Tumor induction in mouse**

The mouse model used in the study is the female F1 progeny of BRCA1 floxed female mice and MMTV-Cre or WAP-Cre male mice. The animals with site specific BRCA1 knockout genotype tissues specific to the expression of the MMTV or WAP promoters are produced by utilizing the CRE-LOX recombination system.

The female F1 chimeras of Wap Cre genetic background were self crossed for five generations, since CRE is under the control of a conditional promoter expressed only during lactation. Thirteen to 15 month old female WAP Cre genetic back ground animals of the F1 generation were used for our study. In contradiction to this, the female F1 experimental animals of the MMTV Cre genetic background of 8 to 10 months old were used for our experiments.

**Randomization of Animals and Drug Administration**

All the F1 mouse strains were grouped in such a way that each cage gets the animals with similar body weight 25- 30 g and the number of animals were 6 per group. Control group (MMTV A or WAP A) were treated with the solvent used for diluting PB. PB was dosed (MMTV PB or WAP PB) alternate days in 25% PEG in normal saline with the concentration of 2 mg/kg body weight. Positive control group were treated with carboplatin (MMTV CN or WAP CN), a known DNA intercalating agent. All these compounds were delivered intraperitonially under the supervision of a veterinary practitioner and under the guidelines of IAEC (IAEC/32/PRIYA/2005). The drug treatment was scheduled for 25 days with body weight evaluation for every seven days.

**Sample Collection**

After the treatment with the compounds all the animals were sacrificed by cervical dislocation. Control and treated mammary tissue were excised and one part of the tissue to be used for protein isolation was snap frozen in liquid nitrogen and stored at -70^o^ C and another portion was fixed with 10% buffered formalin for histopathological analysis. For RNA isolation tissue samples were washed in PBS and placed in All Protect Tissue Reagent from QIAGEN Inc., Valencia, CA stabilized at 4^o^C overnight and stored at -70^o^ C until analysis.

**Preparation of Paraffin Embedded Tissue (PET) Sections**

A part of the normal mammary tissue and mammary tumor excised from the experimental animals was fixed in 10% buffered formalin for 48 h, dehydrated by three passages in acetone (30 min each), two passages in 1:1 mixture of acetone: xylene (25 min each), two passages in xylene (20 min each) and kept immersed in molten paraffin wax overnight. Four microns thick sections were cut out from PET samples using microtome, collected in a poly L Lysine coated charged glass slide and dried for 12 h at 37^o^ C.

**Histopathology by H & E staining**

For histopathologic examination, sections of mammary tumours were prepared and paraffin embedded. They are deparaffinised by keeping the slides at 55^o^C for 10 min and subsequent passages through xylene, rehydrated with graded series of ethanol and distilled water, stained with Meyer’s hematoxylin. Excess stain is washed off and the slides were dipped in differentiation solution (75% alcohol acidified with 1% HCl) for 4 s and counterstained with eosin solution. The sections are rinsed with distilled water, dehydrated with ascending grades of alcohol series, cleared in three changes of xylene and mounted with DPX mountant. The photomicrograph was captured using BX51 Olympus microscope^TM^, Olympus America Inc., Melville, New York and DP 70^TM^ camera with the help of Image Pro Software^TM)^.

**Toxicological analysis**

The animals were observed at least twice a day for any clinical signs: respiratory difficulty, anorexia, diarrhoea and abnormal behaviour. At the end of the experimental period the animals were killed by cervical dislocation and necropsy was conducted. The following organs were selected for histopathology: heart, lung, liver, kidney, spleen, brain and uterus. Routine Haematoxylin & Eosin staining was performed for histological features under the guidance of a veterinary pathologist and photomicrographs of representative lesions were captured.

**Legend for supplementary figures**

**Figure S1. PB is less sensitive to wild type BRCA1 containing normal breast cells.** PB showed less sensitivity to breast cells that are transformed. The differences among the mean values were analyzed using One-way ANOVA followed by bonferroni’s post hoc t- test and p-values were calculated.

**Figure S2. WAP-Cre; BRCA1^Co/Co^ conditional knockout form mammary specific tumors of longer latency.** Mammary tumor formed in WAP-Cre; BRCA1^Co/Co^ of 13 month aged mouse models were analysed using NMR bio imager in which tumors were observed at the 2^nd^ and 3^rd^ mammary glands but the C57 B/L control does not have any tumor.

**Figure S3. PB did not cause toxicity to the experimental mice. A.** Graph representing body weight of mouse treated with PB and CN evaluated every 7 days. **B.** H & E Sections of heart and liver of MMTV-Cre; BRCA1^Co/Co^ (top panel) and WAP-Cre; BRCA1^Co/Co^ animals (bottom panel) with PB, CN and Control group. PB did not cause cardiac toxicity. Mild haemorrhage (*) and occasional degenerated myocadiocytes (thick and thin arrow) were seen in CN treated animals. PB did not induce any significant liver toxicity**.** The histology section of liver from MMTV C and MMTV CN had occasional aggregation of mononuclear cells in the portal tract (thick arrow). Fatty change as well as periportal hepatitis was common. A solitary clear cell focus and microgranuloma was present in MMTV-CN and WAP-CN respectively. The WAP and MMTV group that were treated with PB are annotated as WAP PB and MMTV PB; CN treated group annotated as WAP CN and MMTV CN; solvent treated control group as WAP C and MMTV C respectively.

**
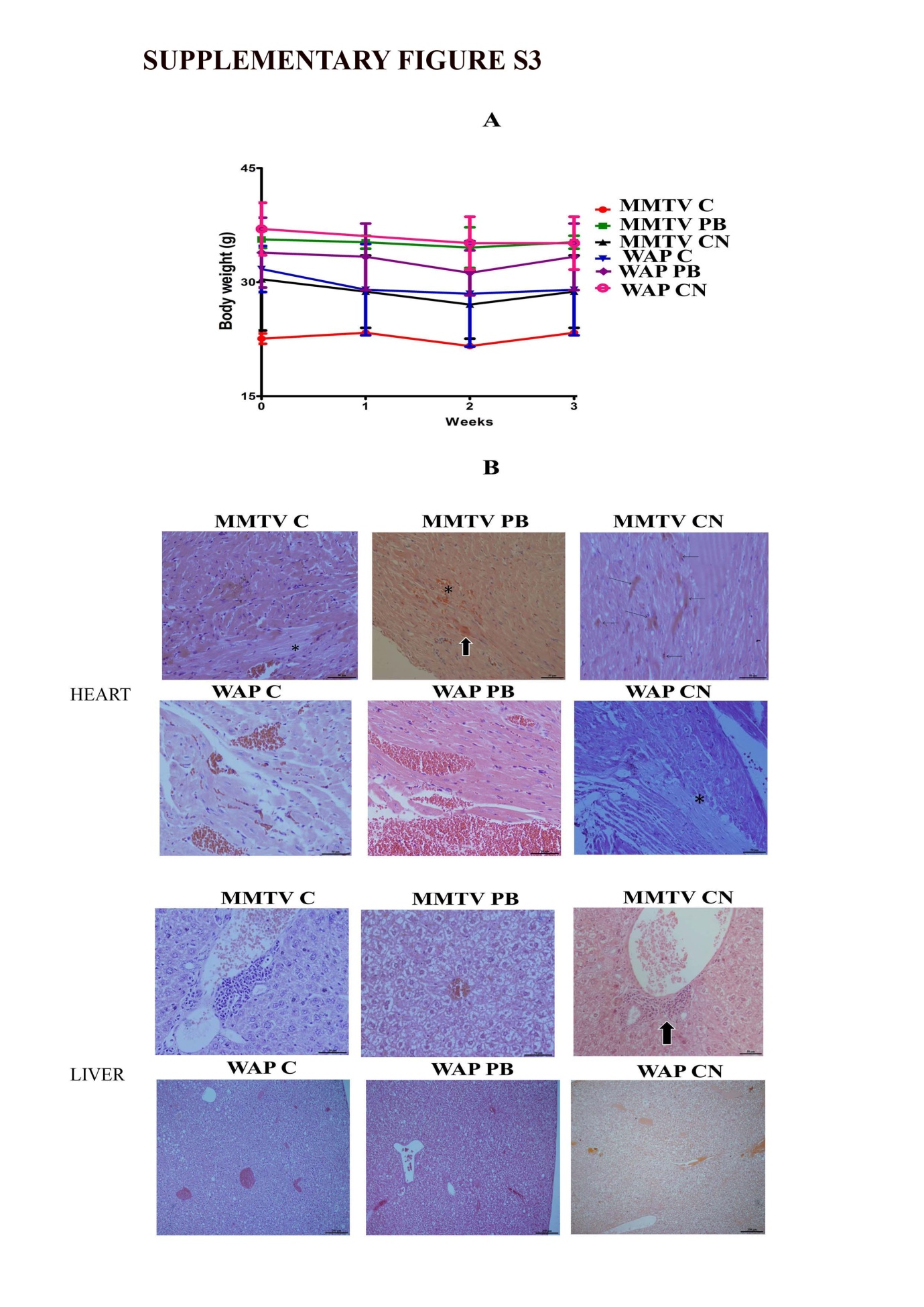
**
